# Musculoskeletal disorders early diagnosis: A retrospective study in the occupational medicine setting

**DOI:** 10.1186/1745-6673-6-1

**Published:** 2011-01-05

**Authors:** John Kulin, MaryRose Reaston

**Affiliations:** 1 Occupational Medicine South, 712 E Bay Ave, Manahawkin, New Jersey, 08050, USA; 2 Insight Diagnostics Inc. 3658 N. Rancho Dr., Las Vegas, Nevada, 89130, USA

## Abstract

Electrodiagnostic Functional Assessment (EFA) objectively evaluates injuries to muscles by incorporating surface electromyography (EMG) to measure myoelectrical signals of muscle groups recorded from up to 18 sensors placed on the skin surface while simultaneously assessing functional capacity at rest and during full range of motion. The evaluation is non-invasive and non-loading and provides measurements in real time. Soft-tissue damage of ligaments, tendons, and muscles, commonly referred to as sprains and strains, has proven to be very difficult to accurately diagnose and assess and represents the highest incidence rate, lost days and medical costs in the workers' compensation system. 100 patients presenting with work-related musculoskeletal injuries exhibiting physical complaints that persisted for at least two consecutive weeks for which no general medical explanation could be established after medical history and exam, were evaluated using EFA in our Occupational Clinic in New Jersey over a 36 month period. The results of this study demonstrated the clinical effectiveness of the EFA as an objective diagnostic aid for identifying and quantifying soft tissue injuries and devising site specific physical therapy treatment regimen to return the injured worker to full duty work release.

## Background

### Impact of Musculoskeletal Disorders on the Workers' Compensation System

The U.S. Department of Labor and Occupational Safety and Health Administration (OSHA) define a musculoskeletal disorder (MSD) as an injury of the muscles, nerves, tendons, ligaments, joints, cartilage and spinal discs. OSHA identifies examples of MSDs to include: Carpal tunnel syndrome, Rotator Cuff syndrome, De Quervain's disease, Trigger finger, Tarsal tunnel syndrome, Sciatica, Epicondylitis, Tendinitis, Raynaud's phenomenon, Carpet layers knee, Herniated spinal disc, and Low back pain. The World Health Organization characterizes work-related MSDs as multifactorial to indicate the inclusion of physical, organizational, psychosocial, and sociological risk factors.

These types of disorders commonly referred to as soft tissue injuries (STI) as well as sprains and strains most often present as injury or pain of the back, neck, shoulder or knee, are a major source of disability. Taken together, they represent the majority of compensable injuries accounting for 29% of total cases [1]. The event or exposure leading to the injury is bodily reaction/bending, climbing, crawling, reaching twisting; overexertion; or repeated overuse [2].

According to OSHA, the average cost per incidence of an MSD is estimated to be $12,000. If surgery is required, the average cost rises to $43,000 per incidence according to the American Society of Orthopedic Surgeons. MSDs cost U.S. industry $15-20 billion in worker's compensation costs with total costs as high as $45-60 billion per year [3].

Although workers compensation claims have steadily decreased at approximately 3% annually for the past two decades, the number and frequency of Permanent Total Disability (PTD) claims has significantly increased since 2005 in part, attributable to the aging of the U.S. workforce. Of equal concern, indemnity & medical costs in workers compensation have continued to increase 9-12% per year, while lost days from work have incurred annual increases of 5-7% [4]. Utilization and pay in Workers Compensation are significantly more for chronic pain-related injuries such as bursitis, carpal tunnel and low back pain than Group Health. Sprains, strains, and tears had the highest incidence rate (51 injuries per 10,000 full-time workers) while carpal tunnel syndrome was the source of the highest median number of days away from work with 27 days [5].

### Diagnostic Challenges of STIs

The standard approach to managing soft tissue injuries is to obtain a medical history and perform a physical examination. Imaging or testing usually is not needed in the early phases of treatment. In most cases, the natural history of an STI condition resolves without intervention. However, in those cases where complaints of pain and disability persist, the Occupational Medicine (OM) provider should adhere to treating the problem within evidence-based medicine (EBM) guidelines.

### Limitations of Standard Diagnostic Tests

While frequently utilized, subsequent diagnostic modalities are, in many cases, not appropriate for assessing soft tissue injuries.

X-ray investigation can be used to assess the possibility of fracture or dislocation; however, in low back pain (LBP) x-ray is rarely indicated. Nerve conduction studies may be used to localize nerve dysfunction, and Electrodiagnosis may help differentiate between myopathy and neuropathy. Magnetic resonance imaging (MRI) and CT scans, while excellent tests to evaluate structure are static and not designed to assess muscle function dynamically (while patient is in motion). In addition, these standard tests all carry a high false positive rate [6]. The results provided by these modalities are subject to different interpretations and may be inaccurate and inconclusive. Despite these shortcomings, 1 in 3 Medicare beneficiaries receive an MRI of their lower back when they complain of pain, rather than trying more recommended - and potentially safer - treatment first, such as physical therapy [7].

Not surprisingly, soft tissue injuries are difficult to diagnose because the above diagnostics are frequently unable to document the presence of pain and loss of function. In many instances, this leads to prolonged duration of disability and lost time, and increased medical costs, based on poorly defined diagnosis and no classification of work category. This can lead to costly misdiagnosis, unnecessary surgery, prolonged treatment periods, and fraudulent claims. In the absence of objective medical evidence, "proof" of a soft tissue injury is typically established through medical records documenting results of medical examinations and the insured's complaints of pain and in cases of litigation, testimony from duelling experts whereby each party presents a medical opinion. The need for accurate, timely and evidence-based diagnosis and treatment for soft tissue injuries is needed to curtail these escalating costs and improve clinical outcomes.

## Non-Work-Related Cost Drivers in Workers' Comp

### Aging of Workforce

In Occupational injuries, the physician's role is to assess the injury, determine causality/work relatedness as well as determine if the injury is acute or chronic pre-existing pathology. This task has become increasingly complex as the workforce gets older, workers develop degenerative pathology that may or may not be the responsibility of the employer. It is estimated that over 57% of the working population would have "abnormal findings" if they were to undergo a lumbar MRI [8].

### Psycho-Social Issues and Symptom Magnification

The concept of probing for and identifying psycho-social issues by OM providers can no longer be ignored. In work-related back and neck pain there is strong evidence that psychosocial variables generally have more impact than biomedical or biomechanical factors [9]. Job dissatisfaction, distress, anxiety and depression are leading predictors of who will file an occupational injury claim [10]. There is a clear link between employee depression, work impairment, and days lost. Employees with depression are 27 times greater work loss likelihood than non-depressed employees [11]. The prevalence of personality disorders in the general population is 10% - 13% [12]. Whereas, in medical-legal claims of chronic disabling neck and back pain patients the prevalence of personality disorders is 70% [13]. Somatisation disorder is a long-term (chronic) condition in which a person has physical symptoms that are caused by psychological problems, and no physical problem can be found [14].

### Accuracy of Patient History

It is important for the OM provider to perform structured and detailed histories. It is not uncommon for patients to forget or deny prior injuries, claims, or operations. One study reported 42% of patients claimed pre-injury status as superior in 15/16 areas tested [16]. Another reported 80% of claimants with spinal/shoulder soft tissue injuries denied pre-existing histories of injuries or operations [17]. Under-reported pre-existing diagnoses preclude the OMP's ability to intervene appropriately and may increase future risks of re-injuries.

In this retrospective analysis of 100 individuals with reported work-related soft tissue injuries, we sought to determine the effectiveness of Electrodiagnostic Functional Assessment (EFA) in diagnosing soft tissue related injuries and to access the impact on outcomes to include claim closure, return to work, and litigation. Patients were initially managed by standard methods including work restrictions, physical therapy, and medications. Patients were referred for EFA testing when physical exam findings had normalized; however, they still reported significant subjective complaints.

## Methods

### Electrodiagnostic Functional Assessment (EFA)

The Electrodiagnostic Functional Assessment (EFA) was utilized to evaluate people who presented with soft tissue injuries. The EFA instrumentation is an FDA 510 K registered Class II Diagnostic Device.

The EFA can objectively determine the nature, acuity, and extent of the injury, the precise location of injury and source of referred pain, the significance of disc pathology and site specific treatment. The EFA is the integration and enhancement of accepted diagnostic tests into one dynamic evaluation. Specifically, EFA incorporates surface electromyography (EMG) to measure myoelectrical signals of muscle groups recorded from up to 18 sensors affixed to the skin surface of underlying muscle groups while simultaneously assessing functional capacity at rest and during full range of motion (ROM). The resulting output is an accurate representation of muscle function and effort. According to the FDA registration, it has false positive rating of +- ten (10) percent. Raw EMG data is analyzed to give a more accurate representation. The limiting factor would be if a packet sample is missed but this is adjusted by reviewing the raw data. Peer reviewed evaluation of clinical and diagnostic utility of surface EMG concluded that it may be useful to detect the presence of neuromuscular disease, allows prolonged recordings of muscle activity from multiple sites simultaneously, and is deemed an acceptable method for recording and quantifying clinically important muscle related activity with the least interference on the clinical picture [18]. In fact, through the concurrent mapping of many co-active muscles and muscle group activity, sEMG as a measure of back function can distinguish individuals with and without LBP with an accuracy of 90%." [19] Functional capacity is measured isometrically, utilizing a strain bar, grip, and pinch instruments incorporating load cells to record performance and effort.

A state-of-the-art ROM apparatus captures full freedom of movement: flexion, extension, rotation, as well as lateral movements of a patient with the sensitivity to monitor muscle group activity dynamically while filtering out positional changes.

### Acute versus Chronic Pathology

EFA can determine the approximate age of an injury by graphical interpretations of myoelectrical activity of muscle groups. Chronic Injuries are characterized by muscle compensation, bilateral changes, absence of the flexion-relaxation response, and bilateral vasoconstriction. Conversely, the presence of muscle spasms and hyperactivity is indicative of an acute injury. The ability to distinguish between acute and chronic pathology provides objective determinations of compensability and apportionment.

### Patient Compliance

EFA can objectively quantify effort and identify patient compliance, malingering, and in pain by recording presence or absence of type II motor recruitment when patient is instructed to perform isometric functional capacity component of the EFA.

## Results

### 100 EFA Cases: Reported Experience and Analysis

Many soft tissue injuries are reported as work related and, consequently, are submitted as worker's compensation claims. Occupational Medicine's (OM) primary goal of injury management is functional restoration and returning the patient to pre-injury status so that the patient is capable of returning to work. The OM physician is best served by treating the patient within EBM guidelines in order to achieve this outcome. Soft tissue injuries are poorly understood and accurate diagnosis has proved elusive. Therefore, correctly diagnosing the problem and its relation to the workplace is imperative. The Electrodiagnostic Functional Assessment (EFA) is an FDA registered diagnostic device specifically designed to objectively diagnose injuries to muscles and connective tissue.

Over a three year period, 103 EFA tests were performed on 100 patients evaluated and treated at Occupational Medicine South, PC an occupational medicine facility in Southern New Jersey. Patients that presented with reported work related soft tissue injuries were initially managed by standard methods including work restrictions, physical therapy and medications. Patients were referred for EFA testing when their physical exam findings had normalized but still reported significant subjective complaints. Three patients that had prior EFA's were evaluated with the EFA at onset of new complaints to compare to baseline.

### Patient Demographics

Of the 100 injured workers that underwent EFA testing 56% were female and 44% male. Patient age ranged from 22 yrs to 66 years. The average age was 43 years. However, 68% of patients were 40 years of age or older. See Table 1.

**Table 1:** Baseline Characteristics of 100 Patients presenting with STIs

*Demographic characteristics*	*Male*	*Female*	*Total*
>Age - mean, years	42.95	44.61	43.24
			
Age category - years			
20 - 29	5	6	11
30 - 39	11	10	21
40 - 49	15	17	32
50 - 59	7	19	26
60 - 69	6	4	10
			
***Distribution of injuries by occupation***	***Male***	***Female***	***Total***

Clerical	1	2	3
Construction	12	2	14
Education	1	5	6
Healthcare	4	27	31
Police/Security	3	1	4
Retail	2	5	7
Sales/Service	2	2	4
Service Technician	12	4	16
Transportation	7	8	15
			
***Site of injury or reported pain***	***Male***	***Female***	***Total***

Cervical	3	4	12
Lower Extremities	3	2	5
Lumbosacral	22	29	51
Shoulder	4	11	15
Thoracic	2	0	2
Multiple Areas	10	10	20

### Site of Injury

The most common site of injury was low back. 65% of all study participants reported injury involving the lower back with over half experiencing pain exclusively at lumbosacral and the remaining involving multiple sites of injury to include low back. Approximately 20% of patients reported shoulder injuries.

### Injuries by Occupation

The most common source for injuries occurred in the Healthcare field. The vast majority were healthcare practitioners (RNs, CNAs) and technical occupations. Most of the injuries were reported as the result of lifting and moving patients. Other common sources accounting for injuries included the Transportation category comprised primarily of drivers of trucks and buses. Cause of injury was either result of vehicular accident or exertional in nature during delivery of cargo followed by construction workers and trade professionals as well service technicians to include auto/boat mechanics, HVAC, utility and apartment superintendents.

### Date of Injury (DOI) and Date of EFA Diagnosis

DOI to date of EFA evaluation ranged from one week to 90 weeks. The average time for EFA test was 16 weeks post injury however, after the removal of outliers, a more accurate average time was approximately 9 weeks. Soft tissue injuries were initially treated with conservative measures such as physical therapy, job modification and medications. The majority of work related soft tissue claims resolved within a 4 to 6 week period without need for further treatment or testing. Patients who did not respond to treatment as expected and/or had physical exam findings which had normalized but still reported significant subjective complaints, were then referred for EFA. The 9 weeks time period is realistic in these patients and practice pattern between initial reporting, treatment, referral for EFA, approval of testing and performance of test.

### EFA Test Results

73% of injured workers were found to have chronic, unrelated pathology, much of it age related degeneration. Since the injury was pre-existing the claim was non compensable and the worker was cleared to return to work. Virtually all of these same workers were found to be non-compliant as well meaning they did not cooperate or malingered when instructed to perform functional capacity and ROM during their EFA evaluation as evidenced by the limited/inappropriate recruitment of type II motor units. Patient Compliance, Malingering and Pain: these results corroborated with the treating physicians diagnosis during initial physical exam. Again, only patients with subjective complaints in the absence of objective findings were given EFA assessment. See Table 2.

**Table 2:** Patient Outcomes

*Outcomes*	*Male*	*Female*	*Total*
Industrially related	17	10	27
Chronic and non-industrially related	27	46	73
Full Duty Work Release	43	55	98
Litigated	1	1	2
			
***Compliance with EFA testing***			

Compliant	17	11	28
Non-complaint	27	45	72
			
***Treatment***			

Physical Therapy (avg. number of sessions)	6.3	5.1	5.9

**Figure 1:**
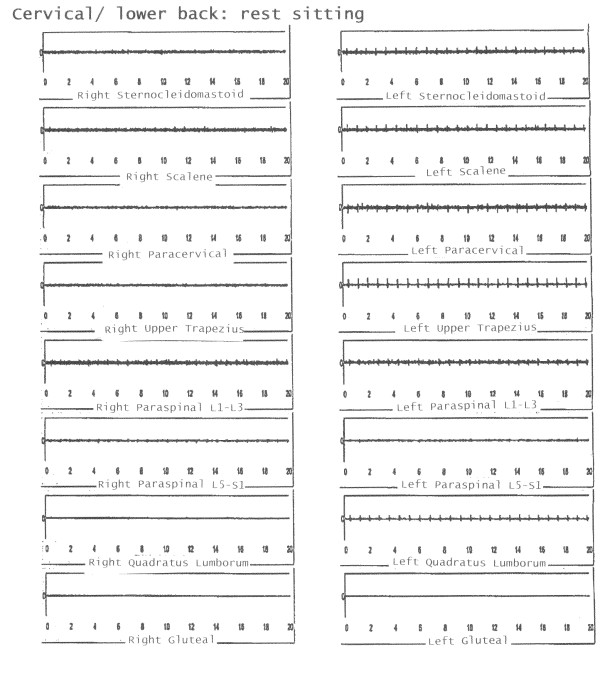
**Acute and Chronic Pathology with lifting**: Acute pathology is demonstrated by frequency response (muscle spasms) chronic pathology is demonstrated by compensation most notably in hamstring muscles.

**Figure 2:**
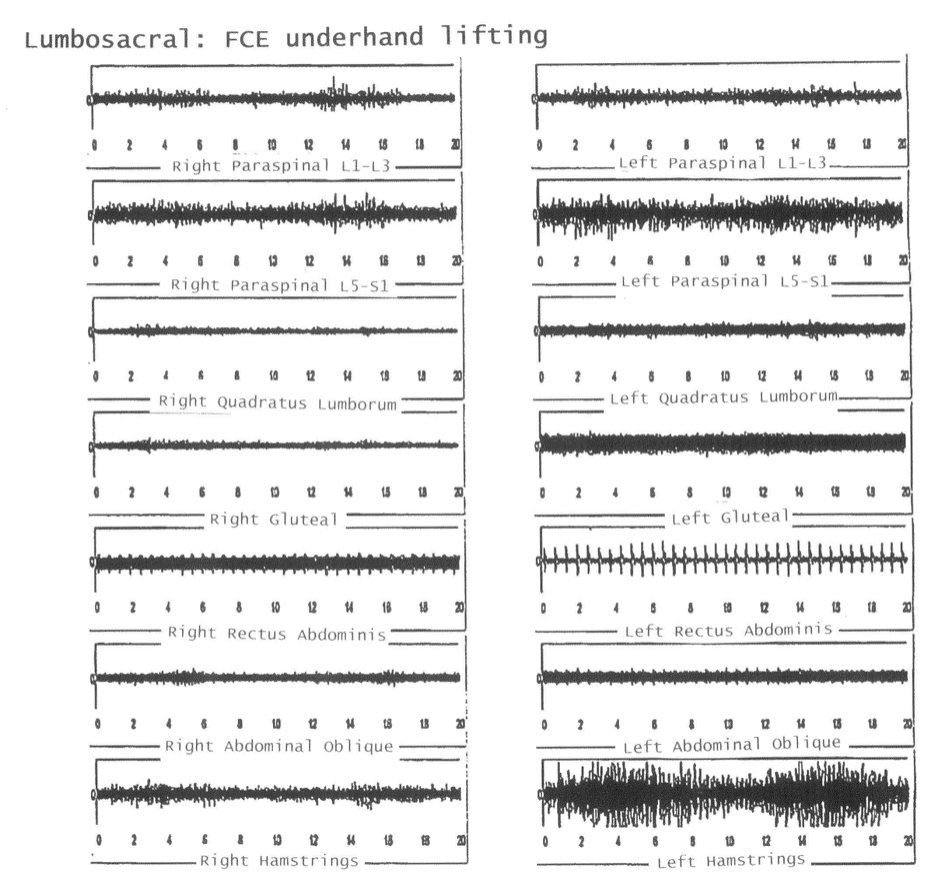
**Chronic Age-related Pathology is shown at rest**. Appropriate EMG readings with ischemic artefact that demonstrates bilateral changes (chronic).

In one instance, the EFA's objective and conclusive data altered the initial diagnosis that the patient did not have significant pathology. EFA results showed significant acute and chronic injury for the worker depicted in Figure 1. The sEMG revealed inappropriate muscle usage, muscle spasms and muscle compensation. This patient was prescribed 12 sessions of site specific PT and was returned to work at MMI pre-injury status. In contrast, Figure 2 depicts the EMG readings of a worker with age-related chronic pathology with absence of acute injury which means this worker did not sustain a work-related injury.

## Discussion

### New Jersey Division of Workers' Compensation

Two patients on Temporary Total Disability (TTD) were stopped after their EFA found no acute compensable pathology. Both patients appealed to New Jersey Division of Workers' Compensation. One is pending and the other was recently settled:

The claimant was involved in an auto accident in May 2008 while operating a school bus with an alleged injury to her cervical and lumbar spine. The MRI revealed positive findings and the claim was accepted as compensable and treatment was authorized. Epidural steroid injections after conservative care failed to alleviate symptoms. After the first injection In March 2009, the claimant alleged numbness and paralysis of the lower extremities and was hospitalized for almost a month as the doctors tried to confirm and diagnose the problem. On discharge, there were no objective findings noted and suggestions for possible psychiatric issues. An EFA was conducted in conjunction with an Independent Medical Evaluation (IME) and found:

• Normal Evaluation

• Inconsistency between the objective findings and subjective complaints

• Objectively non compliant

• Hospitalization not related to or aggravated by date of loss

• Maximum Medical Improvement (MMI) pre injury status with no rateable impairment

Based on the findings of the EFA/IME the carrier denied any further medical or indemnity benefits. The claimant continued allegations for total disability. The claim went before New Jersey Division of Workers' Compensation Judicial Board and was settled for $16 K with a 55 percent savings on the reserve. Of note, all medical payments were prior to the EFA except for the EFA/IME charges.

### Site Specific Treatment

27% of patients had acute pathology and were prescribed site-specific physical therapy (PT) treatment regimen designed to return the worker to MMI with no rateable impairment status and full release to work duty. Recommended PT ranged from 2 to 12 sessions. The average treatment regimen prescribed was 6 PT sessions of muscle-specific therapy. At the conclusion of PT, all workers were released at MMI with no rateable impairment.

## Conclusions

According to the Bureau of Labour Statistics, most occupational injuries are "soft tissue" sprains/strains of the low back, shoulder, neck and knees. Physician directed care based on Evidenced Based Medicine should guide an accurate diagnosis as well as early aggressive conservative intervention. The EFA is an innovative diagnostic aid that is objective, reproducible, definitive, and evidence based. It is a significant in that it can assist an Occupational Medicine provider in objectively assessing the multiple varying subjective complaints and drill down to the soft tissue level to make an accurate diagnosis.

EFA test results affected the course of treatment, improved clinical and functional outcomes, increased patient satisfaction, and decreased dispute litigation. In fact, 98 of the 100 cases resulted in return to maximum medical improvement with no rateable impairment and full release to active duty. Only two percent of the cases were challenged and 98% of the EFA control group returned to their pre-injury job. This paper is a case reference for 100 cases tracked over a three year time period and serves as an illustration of results utilising a new diagnostic aid.

## Competing interests

MR is the president of Insight Diagnostics Inc that provides EFA testing. There are no competing interests for JK.

## Authors' contributions

JK and MR carried out the patient selection, analysis of data and drafting of this manuscript. All authors have read and approved the final manuscript.

## References

[B1] Nonfatal Occupational Injuries and Illnesses Requiring Days Away from Work, 2009 2010 Bureau of Labor Statistics; U.S. Dept of Labor http://www.bls.gov/iif/oshcdnew.htm News Release.

[B2] Injuries, Illnesses, Fatalities and Occupational Safety and Health Definitions 2008 Bureau of Labor Statistics; U.S. Dept of Labor http://www.bls.gov/iif/oshdef.htm

[B3] CFR Part 1910 Ergonomics Program Federal Register/Vol. 64, No 225 1999 Occupational Safety and Health Administration, U.S. Dept of Labor

[B4] DiDonato T Brown D Workers Compensation Claim Frequency Continues Its Decline in 2008 2009 National Council on Compensation Insurance (NCCI) NCCI Research Brief

[B5] Nonfatal Occupational Injuries and Illnesses Requiring Days Away from Work, 2009 2010 Bureau of Labor Statistics; U.S. Dept of Labor http://www.bls.gov/iif/oshcdnew.htm News Release.

[B6] Jensen Brant Ross Zawadzki MN Obuchowski N 1997 331 University of Pittsburgh, NEJM

[B7] 2010 Centers for Medicare & Medicaid Services' (CMS) Hospital Compare MRI and LBP http://www.hospitalcompare.hhs.gov

[B8] Bolden S Davis D Dina T Abnormal MRI scans of the lumbar spine in asymptomatic subjects J Bone Joint Surg 1990 72A 403 409 2312537

[B9] Nachemson AL Jonsson E Neck and Back Pain 2000 Philadelphia, Pa: Lippincott, Williams, and Wilkins

[B10] Bigos, Battie, Spengler. DMA, longitudinal, prospective study of industrial back injury reporting Clin Orthop Relat Res 1992 279 21 34 1534722

[B11] Myette L Depression in the Working Population ACOEM 2009

[B12] Hales RE Yudofsky SCthe American Psychiatric Publishing Textbook of Clinical Psychiatry 2002 Fourth

[B13] Dersh J Prevalence of psychiatric disorders in patients with chronic disabling occupational spinal disorders Spine 2006 31 10 1156 62 10.1097/01.brs.0000216441.83135.6f 16648753

[B14] DeGruy FV Rakel RE The Somatic Patient Textbook of Family Medicine 2007 chap 61 7 Philadelphia, Pa: Saunders Elsevier

[B15] Purcell TB Marx J Somatoform Disorders Rosen's Emergency Medicine: Concepts and Clinical Practice 2006 chap 111 6 Philadelphia, Pa: Mosby Elsevier

[B16] Elliott V Doctors Use New Cues to Get Patient History American Medical News 2003

[B17] Mulford H Claimant-Reported History is Not a Credible Basis for Clinical or Administrative Decision-Making JAMA 2005 293 1644 1652 10.1001/jama.293.13.1644 15811984

[B18] Meekins G American Association of Neuromuscular & Electrodiagnostic Medicine Evidence-Based Review: Use of Surface EMG in the Diagnosis and Study of Neuromuscular Disorders Muscle & Nerve 2008 10.1002/mus.21055 18816611

[B19] Roy DeLuca Emley Rogers 1997 34 4 Memorial Hospital Bedford, MA, JRRD

